# Experimental gel containing bioactive glass-ceramic to minimize the pulp damage caused by dental bleaching in rats

**DOI:** 10.1590/1678-7757-2019-0384

**Published:** 2020-06-08

**Authors:** Marina CARMINATTI, Francine BENETTI, Renato Luiz SIQUEIRA, Edgar Dutra ZANOTTO, André Luiz Fraga BRISO, Antônio Hernandes CHAVES-NETO, Luciano Tavares Angelo CINTRA

**Affiliations:** 1 Universidade Estadual Paulista Faculdade de Odontologia de Araçatuba Departamento de Odontologia Preventiva e Restauradora AraçatubaSP Brazil Universidade Estadual Paulista (UNESP), Faculdade de Odontologia de Araçatuba, Departamento de Odontologia Preventiva e Restauradora, Araçatuba, SP, Brazil.; 2 Universidade Federal de Minas Gerais Faculdade de Odontologia Departamento de Odontologia Restauradora Belo HorizonteMG Brazil Universidade Federal de Minas Gerais (UFMG), Faculdade de Odontologia, Departamento de Odontologia Restauradora, Belo Horizonte, MG, Brazil.; 3 Universidade Federal de São Carlos Departamento de Engenharia de Materiais Laboratório de Materiais Vitreos São CarlosSP Brazil Universidade Federal de São Carlos (UFSCar), Departamento de Engenharia de Materiais, Laboratório de Materiais Vitreos, São Carlos, SP, Brazil.; 4 Universidade Estadual Paulista Faculdade de Odontologia de Araçatuba Departamento de Ciências Básicas AraçatubaSP Brazil Universidade Estadual Paulista (UNESP), Faculdade de Odontologia de Araçatuba, Departamento de Ciências Básicas, Araçatuba, SP, Brazil.

**Keywords:** Bioactive glass, Dental pulp, Hydrogen peroxide, Tooth bleaching, Tooth remineralization

## Abstract

**Objectives:**

This study evaluated if the use of a bioactive glass-ceramic-based gel, named Biosilicate (BS), before, after or mixed with bleaching gel, could influence the inflammation of the dental pulp tissue of rats’ molars undergoing dental bleaching with hydrogen peroxide (H_2_O_2_).

**Methodology:**

The upper molars of Wistar rats (Rattus norvegicus, albinus) were divided into Ble: bleached (35% H_2_O_2_, 30-min); Ble-BS: bleached and followed by BS-based gel application (20 min); BS-Ble: BS-based gel application and then bleaching; BS/7d-Ble: BS-based gel applications for 7 days and then bleaching; Ble+BS: blend of H_2_O_2_ with BS-based gel (1:1, 30-min); and control: placebo gel. After 2 and 30 days (n=10), the rats were euthanized for histological evaluation. The Kruskal-Wallis and Dunn statistical tests were performed (P<0.05).

**Results:**

At 2 days, the Ble and Ble-BS groups had significant alterations in the pulp tissue, with an area of necrosis. The groups with the application of BS-based gel before H_2_O_2_ had moderate inflammation and partial disorganization in the occlusal third of the coronary pulp and were significantly different from the Ble in the middle and cervical thirds (P<0.05). The most favorable results were observed in the Ble+BS, which was similar to the control in all thirds of the coronary pulp (P>0.05). At 30 days, the pulp tissue was organized and the bleached groups presented tertiary dentin deposition. The Ble group had the highest deposition of tertiary dentin, followed by the Ble-BS, and both were different from control (P<0.05).

**Conclusion:**

A single BS-based gel application beforehand or BS-based gel blended with a bleaching gel minimize the pulp damage induced by dental bleaching.

## Introduction

Studies on the bleaching gel containing hydrogen peroxide (H_2_O_2_) have indicated some adverse effects caused by this product.^1-4^ For example, there are reports on the morphological changes in the structure of dental enamel after contact with H_2_O_2_, such as increase in roughness and decrease in hardness.^2,3,5,6^Histochemical changes in dental tissues are observed as a loss of calcium and phosphorus ions in enamel and dentin.^2,5,6^ Decrease in the flexural strength of the teeth was also observed.^3^

These damages occur due to H_2_O_2_ releasing reactive oxygen species (ROS), which react with organic and inorganic molecules.^2,3,5^ ROS can reach the dental pulp due to their low molecular weight^7^ and cytopathological effects are mutation, enzymatic inactivation, protein degradation, and cellular apoptosis or tissue necrosis.^1,4,8-10^ As a consequence, most patients who undergo dental bleaching have postoperative sensitivity characterized by tingling or needles.^11^ This sensitivity can also affect healthy teeth, unlike common dental sensitivity which occurs only in the presence of exposed dentin or cracks in the enamel.^11^

Many therapeutic agents have been tested in an attempt to minimize pulp damage and teeth sensitivity, such as anti-inflammatory substances,^12,13^ antioxidants,^14^ desensitizing^15^ or remineralizing agents.^2,5^ The use of remineralizing agents, such as bioactive glasses and glass-ceramics, is common for these materials present remarkable positive interaction with hard (bone and teeth) and soft tissues.^16-19^ The ability of these materials to form a hydroxycarbonate apatite (HCA) layer on their surfaces *in vivo*, promoting an interface and strong bonds between bone and teeth, can be used to prevent enamel demineralization after dental bleaching.^20^

Biosilicate^®^ (BS), a bioactive glass-ceramic, has been successfully tested in several medical and dental applications.^18,19^ This material can induce the deposition of HCA in dentin tubules^21^, reducing tooth hypersensitivity.^22^ Other studies demonstrated that BS was able to increase microhardness of the enamel after dental bleaching,^23^ in addition to minimizing the demineralization of dental structures caused by bleaching gel.^5^ Furthermore, it showed the ability to reduce the dental sensitivity caused after this aesthetic procedure.^11^ However, the effects on the pulp tissue generated by the use of BS in dental bleaching have not yet been investigated.

The model of rat molars for pulp tissue analysis after dental bleaching was proposed by Cintra, et al.^24^ (2016). This model is easy to standardize specimens, given the difficulty of obtaining a sufficient number of samples for studies in humans.^1,4^ It is thus possible to study different variables in rats, validating the results in humans later, in accordance with ethical principles. Studies concerning cell culture are important to obtain preliminary results, but these studies do not enable analysis on organized tissues. Moreover, vital teeth have dentinal fluid, odontoblast extensions, and antioxidant enzymes^24^ that can minimize the effects of H_2_O_2_ on pulp tissues. Pulp tissue is surrounded by rigid and inextensible dentine walls, which reflects the importance of *in vivo* analysis to assess pulp tissue response to aggressors.^24^

As such, this study evaluates the *in vivo* therapeutic effect of the different application protocols of a bioactive glass-ceramic-based gel, the BS-based gel, on the dental pulp tissue of Wistar rat molars undergoing dental bleaching with H_2_O_2_. Our null hypothesis was that BS-based gel does not minimize the damage that dental bleaching causes to the pulp tissue.

## Methodology

### Development of bioactive glass-ceramic

The bioactive glass-ceramic evaluated in this study, based on a Biosilicate^®^ 48.5SiO_2_–23.75CaO–23.75Na_2_O–4P_2_O_5_ (wt.%) composition, was synthesized using a sol-gel method following a previous study^25^ with some adaptations. The gel preparation involved hydrolysis and polycondensation reactions by stoichiometrically mixing amounts of tetraethoxysilane (TEOS, Si(OC_2_H_5_)_4_ ≥99.0%), triethyl phosphate (TEP, OP(OC_2_H_5_)_3_ ≥99.8%), calcium nitrate tetrahydrate (Ca(NO_3_)_2_·٤H_2_O ≥99.0%), and sodium nitrate (NaNO_3_ ≥99.0%) – all chemicals were provided by Sigma-Aldrich. The product was manually desegregated in an agate mortar after heat treatment at about 700°C to stabilize the gel and form the Na_2_CaSi_2_O_6_ crystalline phase, followed by high energy milling to obtain powders with approximately 500 nm.^26,27^

### Dental bleaching and pulp damage analysis

In total, 60 healthy, 2-months-old male Wistar rats (*Rattus norvegicus, albinus* variation; weighing approximately 280 g) were used in this study. The established sample size was based on previous studies involving the analysis of bleaching gel effects on the pulp tissue of rats^4,28^. During the entire experimental period, the animals were housed in a humidity- and temperature-controlled environment (22±1°C; 55±10% humidity) on a standard light/dark schedule with access to food (Mogiana Alimentos SA, Campinas, Brazil) and water *ad libitum* in collective polypropylene cages (four per cage). Cage bedding was changed at least three times a week. The animals were observed during the entire experimental period. The institutional ethics committee (CEUA00631) approved the experimental protocol. The entire study was conducted according to ARRIVE guidelines.

### Bleaching session and remineralizing protocols

All procedures were performed in an appropriate room in the animal research area. The rats were anesthetized via intramuscular injections of ketamine (87 mg/kg, Ketamina Agener 10%; União Química Farmacêutica Nacional S/A, Embu-Guaçu, São Paulo, SP, Brazil) and xylazine (13 mg/kg, Xilazin; Syntec do Brasil LTDA, Cotia, São Paulo, SP, Brazil). The rats’ upper molars were randomly divided into 6 groups (n=10), with the following treatment being applied to each of them: the control group received the placebo gel; the Ble group received an application of the bleaching gel; the Ble-BS group received an application of the bleaching gel followed by single application of BS-based gel; the BS-Ble group received single BS-based gel application and then the bleaching gel; the BS/7d-Ble group received one BS-based gel application for seven days (one per day) and then received the application of the bleaching gel at day 7; and finally, Ble+BS group received one application of a blend of the bleaching gel with BS-based gel. The randomization of groups was performed by lottery: the upper molars of the right or left side of each rat were drawn for application of treatment and defined by lottery.

After anesthesia, the photo-activation of the resinous gingival barrier (Top Dam; FGM Dental Products, Joinville, SC, Brazil) around the upper molars was performed. Then, 0.01 mL of 35% H_2_O_2_ bleaching gel (Whiteness HP Maxx; FGM Dental Products, Joinville, SC, Brazil), placebo gel (thickener of bleaching gel) or bleaching gel mixed with BS-based gel were applied in the occlusal surface of upper molars using 1.0 mL syringes to standardize the gel volume. Each product remained for a single application of 30 min^28,29^ in each randomized molar. Afterwards, the product was removed with a cotton ball and the molars were rinsed thoroughly with water. The experimental BS-based gel (glass-ceramic) with ~500 nm granulometry was applied using a micro-applicator (Microbrush^®^, Kage Sorensen, Cotia, SP, Brazil) over the occlusal surface of upper molars. The material was rubbed for 30 sec and then remained active for 20 min, similarly to other studies.^2,22^

For the BS-based gel preparation, the BS powder was mixed with distilled water immediately before application, using a ratio of powder weight (mg) to water volume (mL) of 1:10.^21^ The measurement of BS-based gel pH was taken thrice by a digital benchtop pH meter (K39-2014B, Kasvi Equipamentos Laboratoriais, São José do Pinhais, PR, Brazil), which was calibrated using solutions with pH 4.00 and 7.00 before the analysis. The mean value of the three measurements was considered the final pH value,^30^ namely 11.10±0.03. The BS-based gel was prepared and blended with the same volume of bleaching gel, at a 1:1 ratio. After the application of these products, they were removed with a cotton ball and molars were rinsed thoroughly with water. Lastly, the resinous gingival barrier was removed.

### Histology analysis

At the end of 2 and 30 days after the bleaching session,^8,29^ the rats were euthanized with an overdose of the anesthetic solution (150 mg/Kg, Thiopentax; Cristália Produtos Químicos Farmacêuticos LTDA, Itapira, SP, Brazil). For each period, 30 animals were euthanized. The right and left maxillae from each rat were separated, dissected and fixed in a solution of 10% buffered formalin for 24 h. The specimens were decalcified in a 10% ethylenediaminetetraacetic acid solution for 3 months and dehydrated in a graded ethanol series. Then, they were clarified and embedded in paraffin. Six-micron sections were cut in the vestibular-lingual plane and stained with hematoxylin and eosin. The serial histological sections of each specimen were selected from the point where the mesial root of the first molar was seen in its entire longitudinal extension.^4,28^

Histological analysis was performed by a single calibrated blind-rater, under light microscopy (400X, DM 4000 B; Leica, Wetzlar, Germany). The pulp chamber was divided into thirds (occlusal, middle and cervical),^4,8^ and inflammation was scored considering intensity and cell distribution according to the approximate average number of inflammatory cells present in each third of the same specimen, as follows: 1, inflammatory cells absent or negligible in number; 2, mild inflammatory infiltrate (<25 cells per field); 3, moderate inflammatory infiltrate (between 25 and 125 cells per field); 4, severe inflammatory infiltrate (>125 cells per field); and 5, tissue necrosis.^8^ The central area of the pulp chamber was calculated using Leica Qwin Plus software (Leica^®^ Microsystems, Wetzlar, Germany).^28^

### Statistical analysis

Histological data were subjected to the Kruskal-Wallis and Dunn tests. The pulp chamber area values were subjected to the Shapiro-Wilk normality test, and the Kruskal-Wallis and Dunn tests were used. The tests were performed using the software application Sigma Plot (Systat Software Inc., San Jose, CA, USA) at 5% significance (*P*<0.05).

## Results

### Analysis at 2 days after the bleaching session

**Figure 1:**
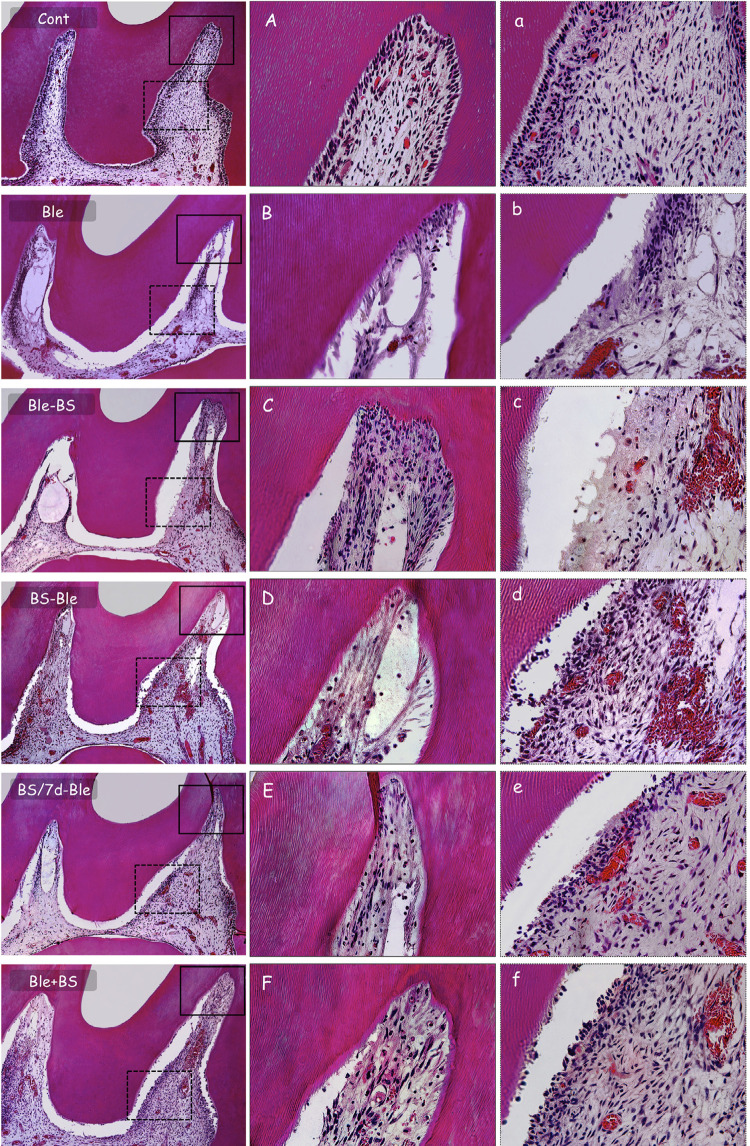
Representative images of the groups at 2 days. Control: panoramic microscopic aspect evidencing normal pulp tissue; (A) pulp horn region and (a) middle third of the coronary pulp with organized pulp tissue. Ble: panoramic microscopic aspect showing disorganization of pulp tissue and severe alterations; (B) pulp horn region with area of necrosis and (b) middle third of the coronary pulp with absence of odontoblastic layer and severe inflammatory infiltrate. Ble-BS: presence of pulp tissue disorganization and severe inflammatory infiltrate; (C) pulp horn and (c) middle third of the coronal pulp tissue with severe disorganization. BS-Ble; BS/7d-Ble; Ble+BS: presence of moderate inflammatory infiltrate in partial tissue disorganization; (D, E, F) pulp horn and (d, e, f) middle third of the coronal pulp with higher tissue organization and presence of inflammatory cell. [100X, 400X; H.E.]

There was no loss of animals in this study. Rats were healthy on days when the hemimaxillae were removed for further processing and histological analysis. The histological aspect of the groups at 2 days can be observed in Figure 1, and scores for inflammation are presented in Table 1.

**Table 1:** Scores and median for inflammatory infiltrate at 2 days in the coronal pulp thirds

Thirds	Score	Groups						P
		Control	Ble	Ble-BS	BS-Ble	BS/7d-Ble	Ble+BS	
Occlusal	1	10/10	0/10	0/10	0/10	0/10	0/10	< 0.001
	2	0/10	0/10	0/10	1/10	2/10	2/10	
	3	0/10	0/10	0/10	7/10	6/10	8/10	
	4	0/10	4/10	6/10	2/10	1/10	0/10	
	5	0/10	6/10	4/10	0/10	1/10	0/10	
	Median*	1^a^	5^b^	4^b^	3^ab^	3^ab^	3^a^	
Middle	1	10/10	0/10	0/10	3/10	2/10	4/10	< 0.001
	2	0/10	0/10	3/10	5/10	7/10	5/10	
	3	0/10	2/10	5/10	2/10	1/10	1/10	
	4	0/10	7/10	2/10	0/10	0/10	0/10	
	5	0/10	1/10	0/10	0/10	0/10	0/10	
	Median*	1^a^	4^b^	3^bc^	2^ac^	2^ac^	2^ac^	
Cervical	1	10/10	0/10	0/10	7/10	6/10	8/10	< 0.001
	2	0/10	2/10	6/10	2/10	4/10	2/10	
	3	0/10	7/10	4/10	1/10	0/10	0/10	
	4	0/10	1/10	0/10	0/10	0/10	0/10	
	5	0/10	0/10	0/10	0/10	0/10	0/10	
	Median*	1^a^	3^b^	2^bc^	1^ac^	1^ac^	1^ac^	

*Different letters in each line indicate a significant difference between groups (P<0.05).

The pulp tissue of the control group showed normality and an intact odontoblastic layer with absence of inflammatory cells. The Ble group had a significant alteration in the pulp tissue, with necrosis and severe inflammation in the occlusal third of the coronary pulp; severe inflammation was also observed in the middle third in most specimens, with a disrupted odontoblastic layer, and the cervical third presented moderate inflammation. In the Ble-BS group, the specimens had disorganization of the pulp tissue in the occlusal third of the coronary pulp, with severe inflammation, and an area of necrosis was observed in some specimens; the pulp had moderate inflammatory infiltrate in the middle third, with tissue disorganization and mild inflammation in the cervical third. Most specimens of the groups that received the application of BS-based gel before the bleaching gel had moderate inflammation and partial disorganization of the pulp tissue in the occlusal third of the coronary pulp; there was mild inflammation in the middle third with higher organization of the pulp tissue and presence of an odontoblastic layer; in the cervical third, most specimens did not present inflammation. The Ble+BS group had mild to moderate inflammatory infiltrate in the occlusal third of coronary pulp, with higher tissue organization and absence of necrosis; in the middle third, this group had mild inflammatory infiltrate in most specimens and the odontoblastic layer was present; there was no inflammation in most specimens in the cervical third, which had organized pulp tissue.

The statistical analysis of scores attributed to the inflammatory infiltrate (see Table 1) at 2 days presented similarity in the occlusal third of the coronary pulp to the Ble and Ble-BS groups (*P*>0.05), which had significant inflammation compared to that of the Ble+BS group (*P*<0.05); no difference was found between the groups that received BS-based gel before the bleaching gel and the other bleached groups (*P*>0.05); the groups that received BS-based gel before the bleaching gel and Ble+BS groups were similar to the control group (*P*>0.05). In the middle and cervical thirds, the inflammation in the Ble-BS group was similar to that in the Ble group (*P*>0.05) and significantly higher than that of the control and other bleached groups (*P*<0.05). Groups with the application of BS before or blended with the bleaching gel had inflammatory response without difference to the control group (*P*>0.05).

### Analysis at 30 days after the bleaching session

**Figure 2:**
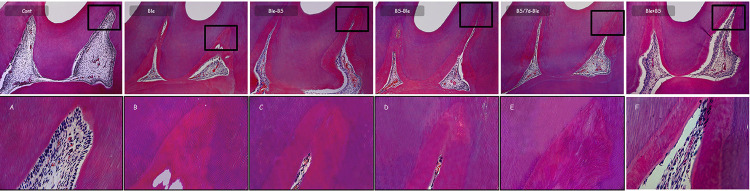
Representative images of the groups at 30 days. Control: cellular and tissue organization (A). Ble (B), Ble-BS (C), BS-Ble (D), BS/7d-Ble (E), Ble+BS (F): absence of inflammation and deposition of tertiary dentin. [100X, 400X; H.E.]

The histological aspect of the groups at 30 days in Figure 2 showed absence of inflammatory cells in their specimens, with organized pulp tissue and an intact odontoblastic layer throughout the pulp chamber. However, the groups that received the bleaching gel presented the deposition of tertiary dentin in a large part of the pulp chamber.

The central area value of each group at 30 days is presented in Table 2. The deposition of tertiary dentin was higher in the Ble group compared to the control and Ble+BS groups (*P*<0.05). The Ble-BS group was different from the control group (*P*<0.05), and no significant difference was found among the other groups (*P*>0.05).

**Table 2:** Mean of the central area of the pulp chamber at 30 days

Groups*	Mean (10^**5**^)	Max-min value (10^**5**^)	% Reduction	n	P
Control^a^	22.1	28.3 – 16.1	0.0	10	< 0.001
Ble^b^	6.8	10.0 – 3.5	69.2	10	
Ble-BS^bc^	11.9	20.4 – 4.3	45.9	10	
BS-Ble^ab^	13.9	24.0 – 6.00	37.0	10	
BS/7d-Ble^ab^	14.3	17.5 – 12.1	35.2	10	
Ble+BS^ac^	15.4	18.3 – 12.8	30.3	10	

*Different letters in each column indicate a significant difference between groups (P<0.05).

## Discussion

This study analyzed if different application protocols of a BS-based gel could minimize the damage caused in the pulp tissue of bleached teeth, and it revealed that the BS-based gel reduced the inflammatory process in the pulp tissue only when applied before or blended with the bleaching gel. Therefore, the null hypothesis that BS-based gel does not minimize the damage that dental bleaching causes to the pulp tissue was rejected.

In this study, a high-concentration bleaching gel was used and it caused severe damage to the pulp tissue, such as necrosis. These results were observed in previous studies^4,7,28,29^ and they were similar to those found in human mandibular incisors.^1^ Severe toxic effects caused by bleaching gels have also been observed in pulp cell cultures, decreasing cellular metabolism and causing morphological changes.^9,10^ The anti-inflammatory substances and low level laser therapy used have been tested and can promote reduction of pulp damage after dental bleaching, but the results are not conclusive.^9,14^ The reduction of the bleaching gel concentration used in in-office bleaching is also an option to minimize the damage.^7,8,31^ However, this product requires repeated sessions containing lower H_2_O_2_ concentration,^6^ a procedure which may also increase the damage to pulp tissue.^4^ However, BS was effective in reducing the post-operative sensitivity reported by patients with bleached teeth, besides minimizing the effects of the bleaching gel on mineralized dental tissues.^11,23^

Previous studies showed that formulations of 16% carbamide peroxide bleaching gel containing BS were relevant to improve the enamel microhardness after bleaching.^23^ The BS powder, with granulometry inferior to 10μm, could induce the deposition of HCA in exposed dentinal tubules^21^ and its use immediately after bleaching reduced the demineralization that bleaching products cause,^5^ thus preventing the exposure of dentinal tubules. BS powder was also indicated for the treatment of dentin hypersensitivity, with highly positive results,^22^ and its applications after dental bleaching significantly reduced post-operative sensitivity of patients with bleached teeth.^11,23^

BS and other similar glass-ceramic materials also presented high positive-interaction with tissues, stimulating tissue regeneration.^17,18,19^ Thus, this study investigated if the application of BS-based gel after bleaching gel could positively affect the pulp cells, stimulating tissue recovery. Our results indicate that BS was not able to induce the healing of pulp tissue after damage from the dental bleaching. It may be that BS did not have capacity to penetrate the pulp tissue to promote sufficient cellular stimuli, even with a sample produced via sol-gel that enables the creation of powder with finer particles and higher reactivity. Thus, this result corroborates the idea that the reduction of sensitivity in bleached teeth after the use of BS originated from the sealing of the dental surface, which prevents or minimizes the external stimuli that could stimulate the inflamed pulp, increasing the sensation of pain.^22^

The sol-gel system did not allow precise control of the particle sizes. It is not yet known if there is significant influence of the particle size and/or the synthesis route applied for BS in this particular application. Tirapelli, et al.^21^ (2010) showed that the size of BS particles influences the penetration capacity in dentinal tubes and that these particles possibly decrease over time in contact with water. Our study did not identify whether the particles in BS-based gel were sufficient to penetrate the dentinal tubes of molar rats and reach the pulp. Moreover, this study applied BS-based gel in teeth immediately after its preparation, minimizing the reduction of particle size by contact with liquid.

The use of BS-based gel before bleaching had positive results in the inflammatory process. The BS is constituted by the Na_2_CaSi_2_O_6_ crystalline phase,^18,19^ which allows a higher local concentration of Na^+^ and Ca^2^ released to the dental surface, maintaining a slightly alkaline medium. We observed that BS-based gel used in this study actually had alkaline pH. This may have contributed positively to our results by minimizing the acidification caused by the H_2_O_2_.^10^ Furthermore, the alkaline medium is a favorable condition for the formation of an HCA layer^18,19,25,32^ on the dental surface, reinforcing and protecting such surface. Moreover, the BS ability to improve the hardness and ionic concentration of dental enamel^5^ can favor a reduction in the penetration of H_2_O_2_ to the pulp tissue, which should be evaluated.

Various BS-based gel applications before the bleaching did not show better results compared to a single application. On the contrary, during the treatment of dentine hypersensitivity using BS, more applications enabled greater reduction of hypersensitivity.^22^ The differences in the experimental model should be considered, since only a single immediate application may have been sufficient for changing the surface of rats’ molars, and additional applications had no significant effects. The difference in particle size should also be considered (~500 nm in this study *versus* 1 – 20 μm in a previous study^22^) and in the synthesis procedure to produce the BS (sol-gel in this study *versus* traditional glass-ceramics route in a previous study).^22^ These parameters influence the reactivity of material. Moreover, the same authors also observed that 20 min of a single application of the product led to reduction in the pain response due to the stimulation of air from the triple syringe, indicating the quick action of BS.

BS-based gel associated with the bleaching gel presented the best effect on the pulp tissue. This blend can reduce H_2_O_2_ concentration, which can influence the results of this study. As the blend was obtained with the same proportion of BS-based gel and bleaching gel, it may have a concentration of 17.5% H_2_O_2_. Studies have shown that this H_2_O_2_ concentration (obtained by blending the bleaching gel with distilled water) has bleaching efficacy similar to 35% H_2_O_2_, promoting lower cytotoxicity.^33-35^

However, a previous study demonstrated that the use of bioglass was associated with bleaching gel maintains the ionic concentration in dental tissues, a fact that supports the integrity of the tissues during the bleaching.^2^ The study also showed that the bleaching efficacy did not change when associating the bioglass with the bleaching gel. Thus, the benefits of blending the bleaching gel with the BS-based gel may be greater than just reducing its H_2_O_2_ concentration. These data are favorable for the use of BS blended with bleaching gel.

After 30 days of the bleaching session, the pulp tissue of all groups was organized and a large amount of tertiary dentin was observed in the bleached groups. Thus, it was possible to verify that the pulp tissue is able to recover from the damage caused by H_2_O_2_. Other studies also demonstrated the reversal of such damage.^8,29,31^ Notably, this tertiary dentin formation indicates the aging of the pulp tissue impairing its ability to defend against new aggressors.^28,31,36^

In this study, the BS-based gel before or blended with bleaching gel enabled a significant reduction in deposition of tertiary dentin, and once again, showed the beneficial effect of using this blend. Thus, the remineralizing products previously used to recover lost ions from dental tissues after contact with H_2_O_2_ can also be applied to prevent the pulp inflammation and to reduce the tertiary dentine production of this tissue. Consequently, postoperative sensitivity can be reduced. Moreover, when blended with bleaching gel, the results are better, besides reducing the clinical time.

A limitation of this study is the fact it has been performed on rat teeth, and thus, the results cannot be extrapolated directly to results in humans. However, rat molars have similar anatomical, histological, and physiological characteristics with human teeth.^24^ Furthermore, standardized animal studies allow different groups to be compared without the influence of other variables, showing in this case the significant effects of mixing BS-based gel with bleaching gel.^24^

However, this was the first study using a BS-based gel blended with a bleaching gel with high H_2_O_2_ concentration, and further evaluation is required before its clinical use. Moreover, this study highlights that the effects in pulp tissue should be priority when performing procedures on the tooth surface, since some dental materials may cause severe alterations in this tissue,^4,31^ as occurs with dental bleaching.^2,3,9,37^ This damage alters the pulp tissue even after a long period.^28,36^

## Conclusions

A single BS-based gel application beforehand or BS-based gel blended with bleaching gel can minimize the pulp damage induced by dental bleaching.
